# Novel *SMARCA2* :: *DDIT3* Fusion in Primary Subcutaneous Myxoid Liposarcoma due to an Unusual Unbalanced Chromosomal Translocation

**DOI:** 10.1002/gcc.70089

**Published:** 2025-10-18

**Authors:** Ahmed Shah, William R. Sukov, Kevin Halling, Lukas Marcelis, Andre Oliveira

**Affiliations:** ^1^ Department of Laboratory Medicine and Pathology Mayo Clinic Rochester Minnesota USA; ^2^ Department of Laboratory Medicine and Pathology University of Alberta Edmonton Alberta Canada; ^3^ Department of Pathology UZ Leuven Belgium

**Keywords:** DDIT3, fluorescent in situ hybridization, myxoid liposarcoma, next generation sequencing, SMARCA2

## Abstract

Myxoid liposarcoma (MLS) accounts for 20%–30% of all liposarcomas, with most cases harboring the fusion gene *FUS::DDIT3*, while approximately 5% exhibit the *EWSR1::DDIT3* fusion. We report the case of a 26‐year‐old male patient with a right upper arm mass. The tumor displayed the classic histological features of MLS, including small spindle/ovoid cells, variable univacuolated lipoblasts, and a prominent myxoid stroma with delicate arborizing vasculature. Despite these characteristic features, fluorescence in situ hybridization (FISH) revealed no apparent rearrangement of the *DDIT3* locus. Next‐generation sequencing (NGS) identified a novel fusion transcript in which *SMARCA2* exon 4 was fused in‐frame with *DDIT3* exon 2. Chromosomal microarray analysis demonstrated the unbalanced nature of the rearrangement, with partial deletions of 0.243 and 0.176 Mb flanking the centromeric end of the *DDIT3* locus on 12q13.3 (which also included *GLI*) and disrupting the *SMARCA2* locus on 9p24, respectively. The resultant chimeric fusion protein is predicted to lack the *SMARCA2* DNA‐binding domains while retaining the *DDIT3* leucine zipper dimerization domain. These findings indicate an unusual and complex rearrangement, leading to the recruitment of a novel *DDIT3* partner gene. Moreover, they emphasize that the functional aspects of myxoid liposarcoma fusion genes depend on the retention of the key *DDIT3* domain. Finally, this case illustrates how classic morphology can appropriately trigger reflex molecular analyses, which may, in turn, uncover novel fusion genes or other molecular alterations.

## 1 Introduction

Myxoid liposarcoma (MLS) is a malignant adipocytic neoplasm accounting for approximately 30% of liposarcomas, typically affecting young adults [1]. It most commonly arises in the deep soft tissues of the extremities and displays a distinctive histologic pattern characterized by uniform spindle‐to‐ovoid cells in a myxoid matrix with arborizing vasculature and scattered lipoblasts. Although primary subcutaneous variants have also been reported [2]. Molecularly, MLS is defined by pathognomonic fusions involving the *DDIT3* gene on 12q13.3. The most frequent rearrangement is *FUS*::*DDIT3*, present in 90%–95% of cases, followed by *EWSR1*::*DDIT3* fusions in the remaining minority [3, 4].

These fusions are usually detectable by fluorescence in situ hybridization (FISH) using break‐apart probes for *DDIT3* and its partners. However, diagnostic pitfalls have been increasingly recognized in the context of cryptic or complex rearrangements. Recent studies have shown that MLS cases with canonical morphology may yield false‐negative FISH results due to occasional unbalanced translocations, insertions, or fusion events that escape detection by standard probe design. For example, Ibstedt et al. described an *EWSR1*::*DDIT3* fusion in MLS that was missed by FISH and cytogenetics but identified by RNA sequencing, highlighting the need to be mindful of these false‐negative studies and knowing when to utilize advanced molecular techniques in ambiguous cases [5].

Here, we report a diagnostically challenging case of morphologically classic MLS with negative FISH for *DDIT3* rearrangement, in which targeted RNA‐based next‐generation sequencing (NGS) uncovered a novel *SMARCA2*::*DDIT3* fusion.

## 2 Case

A 28‐year‐old Caucasian male presented with a slowly enlarging, painless subcutaneous mass in the right upper arm, initially suspected to be a lipoma based on clinical examination. Due to progressive growth, surgical excision was performed. Macroscopically, the resected specimen measured 4.8 cm and consisted of a well‐circumscribed, encapsulated mass with a homogeneous, glistening red‐tan to yellow‐tan cut surface.

**FIGURE 1:**
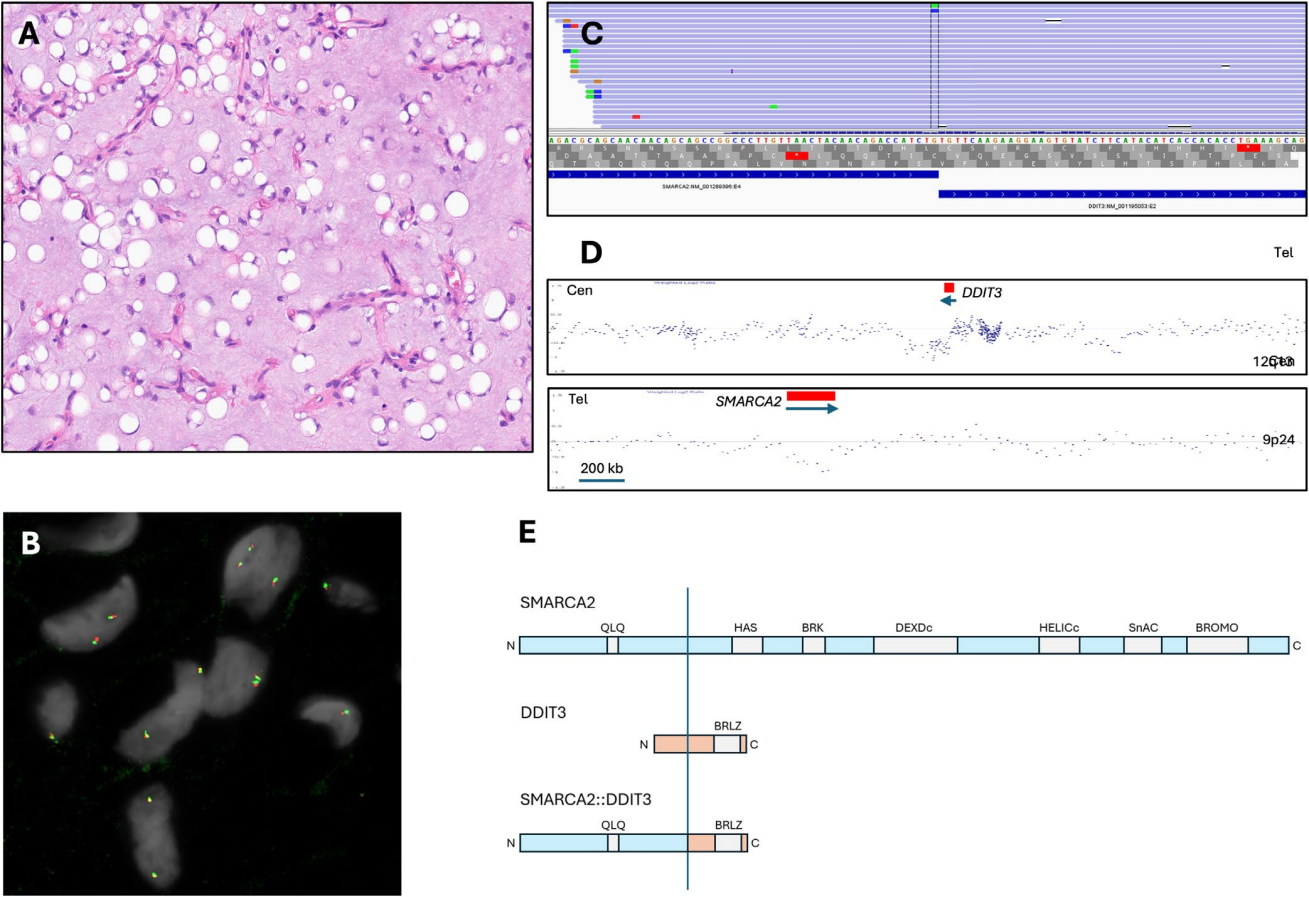
Histologic, immunophenotypic, and molecular features of myxoid liposarcoma with *SMARCA2::DDIT3* fusion. (A) Medium‐power view showing abundant myxoid stroma with delicate, arborizing capillary vasculature and scattered signet‐ring lipoblasts (H&E, ×100). (B) Interphase fluorescence in situ hybridization (FISH) using a dual‐color break‐apart probe targeting the *DDIT3* locus at 12q13.3 [5′ (green)/3′ (red)]. (C) RNA‐based next‐generation sequencing demonstrates split reads supporting a fusion between exon 4 of *SMARCA2* and exon 2 of *DDIT3*. The blue track below illustrates alignment of fusion reads across the junction. (D) Chromosomal microarray plots. Top: Segmental deletion in the region flanking *DDIT3* on chromosome 12q13.3. Bottom: Interstitial deletion involving *SMARCA2* on chromosome 9p24.3. Both deletions are indicated by log2 ratio shifts (Tel, telomeric side, Cen, centromeric side, arrows, direction of transcription; red bars, genes lock). (E) Diagram of the predicted fusion protein *SMARCA2*::*DDIT3* (QLQ, Gln, Leu, Glc protein interaction motif; HAS, helicase domain; BRK, transcription/chromo domain helicase; DEXDc, DEAD‐like helicase domain; HELICc, helicase superfamily C‐terminal domain; SnAC, Snf2‐ATP coupling, chromatin remodeling complex domain; BROMO, bromo domain; BRLZ, basic leucine zipper domain; N, N‐terminal; C, C‐terminal; vertical line indicates approximate location of the breakpoint).

Histologic examination revealed classic features of MLS, including abundant myxoid stroma, delicate arborizing (“chicken‐wire”) vasculature, and numerous univacuolated signet‐ring lipoblasts (Figure 1A). The tumor cells were ovoid to spindle‐shaped with minimal cytologic atypia. Immunohistochemically, the neoplastic cells showed patchy positivity for S100 protein and were negative for desmin, smooth muscle actin (SMA), and CD34.

FISH analysis using a break‐apart probe for the *DDIT3* locus (Abbott Molecular, Des Plaines, IL, USA), performed according to established methods, showed apparently no rearrangement (Figure 1B) [6]. However, targeted RNA‐based NGS using the same methodology as the described SARCP study [7], followed by confirmatory RT‐PCR identified a novel in‐frame *SMARCA2*::*DDIT3* fusion, involving exon 4 of *SMARCA2* and exon 2 of *DDIT3* (Figure 1C). Single nucleotide polymorphism (SNP) chromosomal microarray (OncoScan FFPE Assay Kit; Thermo Fisher Scientific; Santa Clara, CA), performed as previously published, uncovered the unbalanced nature of the rearrangement with deletions around both loci (Figure 1D) [8]. Based on the characteristic morphology and identification of a pathogenic *DDIT3* fusion, the lesion was diagnosed as MLS. The tumor corresponded to FNCLCC grade 1 (low grade).

The tumor was completely resected with negative margins, and no re‐excision or adjuvant therapy was performed. The patient was last seen 6 months after the initial diagnosis, with no evidence of disease at that time, but has since been lost to follow‐up.

## 3 Discussion

This case expands the molecular spectrum of MLS by identifying a novel *SMARCA2::DDIT3* fusion gene in a tumor with classic histologic features but a negative FISH result for *DDIT3* rearrangement. *SMARCA2*, also known as *BRM*, encodes a catalytic subunit of the SWI/SNF chromatin remodeling complex [9]. It interacts with several transcription factors and other DNA‐binding proteins and is involved in chromatin structural modification in the epigenetic regulation of gene expression [9]. To our knowledge, *SMARCA2* has not previously been reported as a fusion partner in MLS. While the predicted fusion protein includes only the QLQ (Gln‐Leu‐Gln) motif of SMARCA2, which is involved in protein–protein interactions, it retains the entire protein‐coding sequence of DDIT3 (Figure 1E), which includes the basic leucine zipper (BRLZ or bZIP) domain required for DNA binding and dimerization—a feature consistently preserved across known pathogenic *DDIT3* fusions [3, 10]. This finding further supports that the C‐terminal transcriptional domain of DDIT3 is critical for mediating its oncogenic potential.

The discrepancy between the fusion‐positive RNA result and the negative *DDIT3* FISH prompted further genomic investigation. Chromosomal microarray analysis revealed a more complex picture with interstitial deletions around fusion partner loci—a segmental heterozygous deletion on chromosome 9p24 partially overlapped the *SMARCA2* locus, and a heterozygous second deletion on chromosome 12q13.3 flanked the *DDIT3* region. This region lacked probe coverage directly within *DDIT3*, limiting precise localization of the breakpoints. Importantly, the *DDIT3* FISH probe set spans ~663 kb telomeric and ~700 kb centromeric to the gene, leaving a ~134 kb unprobed interval that includes *DDIT3*. Rearrangements within this gap can evade detection, leading to false‐negative FISH results. Furthermore, the adjacent *GLI1* gene—centromeric to the 3′ end of *DDIT3*—was preserved, suggesting that the deletion more likely involved the 5′ regulatory or promoter region of *DDIT3*. This is consistent with the observed fusion transcript, in which *SMARCA2* exon 4 is joined to *DDIT3* exon 2, confirming preservation of the functional *DDIT3* domain.

This case illustrates several important diagnostic principles. First, FISH assays may fail to detect cryptic or complex rearrangements, particularly when both breakpoints occur within the region spanned by the break‐apart probes [5, 11]. Second, chromosomal microarrays may have limited resolution in probe‐sparse regions, and interpretations must be approached with caution—especially when no probes directly interrogate the gene of interest. In such cases, RNA‐based NGS is uniquely positioned to resolve uncertainty by directly identifying functional fusion transcripts, regardless of the underlying genomic structural complexity.

In summary, this case identifies *SMARCA2* as a previously unreported *DDIT3* fusion partner in MLS and highlights the diagnostic challenges posed by cryptic rearrangements. It reinforces the essential oncogenic role of the *DDIT3* transcriptional domain and underscores the critical role of RNA‐based testing in resolving molecular ambiguity. For tumors with classic morphology but negative initial testing, reflexive RNA sequencing not only clarifies diagnosis but may also uncover novel fusion events that broaden our understanding of tumor biology.

## Ethics Statement

This case report was conducted in accordance with institutional guidelines. Ethics approval was not required in accordance with Mayo Clinic policy for single‐patient case reports.

## Conflicts of Interest

The authors declare no conflicts of interest.

## Data Availability

The data that support the findings of this study are available from the corresponding author upon reasonable request.
